# Effects of intermittent fasting on quality of life tolerance of chemotherapy in patients with gynecological cancers: study protocol of a randomized-controlled multi-center trial

**DOI:** 10.3389/fonc.2023.1222573

**Published:** 2023-07-19

**Authors:** Mona Wanda Schmidt, Walburgis Brenner, Susanne Gebhard, Marcus Schmidt, Susanne Singer, Lina Weidenbach, Harriett Hahn, Diana Puzankova, Bettina Blau-Schneider, Antje Lehnert, Marco Johannes Battista, Katrin Almstedt, Anja Lütkemeyer, Markus Philipp Radsak, Aline Mähringer-Kunz, Slavomir Krajnak, Valerie Cathrine Linz, Roxana Schwab, Boris Gabriel, Annette Hasenburg, Katharina Anic

**Affiliations:** ^1^Department of Gynecology and Obstetrics, University Medical Center of Johannes Gutenberg University of Mainz, Mainz, Germany; ^2^Department of Gynecology and Obstetrics, Management of the Scientific laboratories, University Medical Center of Johannes Gutenberg University of Mainz, Mainz, Germany; ^3^Division of Epidemiology and Health Services Research, Institute of Medical Biostatistics, Epidemiology and Informatics (IMBEI), University Medical Center of Johannes Gutenberg University of Mainz, Mainz, Germany; ^4^Department of Obstetrics and Gynecology, St. Josefs Hospital Wiesbaden Academic Teaching Hospital, Wiesbaden, Germany; ^5^IIIrd Department of Medicine, Medical Center of the Johannes Gutenberg-University of Mainz, Mainz, Germany; ^6^Department of Radiology, University Medical Center of Johannes Gutenberg University of Mainz, Mainz, Germany

**Keywords:** intermittent fasting, quality of life, fatigue, gynecological cancers, chemotherapy

## Abstract

Fatigue is a very common side effect during intravenous chemotherapy. Unfortunately, only few effective therapeutic options are available, mostly based on daily activity. In our pilot trial we were able to demonstrate that intermittent fasting can reduce fatigue in healthy people, thus we aimed to assess the effects of the fasting dietary on quality of life during chemotherapy in patients with gynecological cancer, especially on the domain of fatigue. The IFAST trial is designed as a prospective, randomized-controlled, multi-center trial. Participation will be offered to women with gynecological cancers (breast cancer, ovarian cancer including peritoneal and fallopian tube cancers, endometrial cancer and cervical cancer) who are planned to receive intravenous chemotherapy for at least three months. Eligible patients will be randomized 1:1, stratified by tumor type and study center. Primary endpoint is the difference in mean change in fatigue, assessed with the Functional Assessment of Chronic Illness Therapy-Fatigue Scale (FACIT- FS^©^). Exploratory secondary endpoints will include general Quality of Life impairment, tolerance of chemotherapy, immunological changes, peripheral cell damage in blood cells, as well as tumor response to chemotherapy. There is new evidence that prolonged fasting periods of 46-96 hours during chemotherapy can positively influence the quality of life during chemotherapy. However, these fasting regiments are not feasible for many patients. Intermittent fasting could be a feasible (manageable) option for many patients to actively improve their quality of life and tolerance to chemotherapy and possibly even enhance the effectiveness of chemotherapy.

**Trial Registration:**

https://drks.de, identifier DRKS00031429.

## Introduction

In the 20^th^ century, researchers found that caloric restrictions prolonged the life span of rodents and reduced age-related diseases (1). Since then, therapeutic fasting has become popular in multiple medical disciplines and its positive effects on health have been documented in many preclinical and clinical trials (2). Among others, intermittent fasting (IF) as a form of time-restricted feeding has demonstrated its capability to reduce insulin resistance (3), oxidative stress and systemic inflammation (4, 5) as well as to increase neuronal stress resistance (6). Despite or maybe due to its wide establishment in the fitness and medical community, the term IF is often mistakenly used interchangeably with short-term fasting (STF) or periodic-fasting. It is characterized by a recurring switch between fasting periods (0-calory or subtotal calory intake) and eating periods (often no caloric limit or specific food restrictions). The lack of a commonly accepted definition to differentiate between the wide variety of fasting regiments needs to be kept in mind, when addressing therapeutic fasting. In a current paper, Longo et al. define IF as “an eating pattern in which short periods of fasting (16 to 48 hours) and eating periods (8 to 12 hours) are alternated” (7). In line with this definition, in our trial we define IF as a period of total calory restriction of at least 16 hours, followed by an average eating period of 8 hours, during which food intake is allowed (16:8h-IF). In contrast to this, STF or periodic fasting are often associated with longer fasting periods of around 60-72h up to multiple days (7, 8).

Besides its quantitatively measurable long-term health benefits, 16:8h-IF has recently been shown to improve quality of life (QoL) and especially the domain of fatigue in a generally healthy study population (9). These results are of great importance not only for mainly healthy people suffering from psychological or physical stress for instance due to shift work, but also for patients with cancer undergoing anti-neoplastic therapies. Especially chemotherapy (CHT) is often associated with a drastic reduction in QoL and increased fatigue, with around 62% of patients suffering from fatigue during treatment (10) and about one third still suffering from it half a year later (11). While some side effects of CHT can be sufficiently treated with modern drugs (e.g. antiemetics, analgesics), there is still a tremendous lack of treatment options for chemotherapy-induced side effects including fatigue (12). Recently, STF has shown to increase QoL and tolerance to CHT in 34 patients with ovarian and breast cancer (13) and the results of a randomized-clinical multi-center trial on STF during CHT are eagerly awaited (8). In contrast, in the DIRECT trial, de Groot et al. found no effect for a fasting-mimicking diet on QoL during CHT in 131 women with breast cancer compared to a regular diet. However, it has to be noted that solely 20% of patients in the fasting-mimicking diet group completed the trial according to the protocol guidelines. The authors report a dislike of distinct components of the fasting-mimicking diet, perhaps induced by the CHT as the main reason for non-compliance and premature discontinuations in the intervention group (14).

IF could be a promising alternative to fasting-mimicking diets, due to its non-calory restrictive and non-ingredient restrictive eating period, which offers freedom in food choice to all patients. The everyday compatibility of IF has demonstrated a high compliance of 90% in a trial conducted by our group (9).

In preclinical trials, fasting has furthermore shown synergistic antitumor activity with antineoplastic therapies (15–17). Partly, this effect is thought to be due to the reduced levels of insulin-like growth factor-1 (IGF-1) concentrations during fasting, which led to a downregulation of the mTOR, RAS and AKT pathways and consequently to an increased autophagy of healthy cells and a shifting of the energy resources towards cell repair and protection mechanisms (17–19). Cancer cells however often lack these regulatory mechanisms. This protection of healthy cells but not cancer cells against stressors including CHT has been described as “differential stress resistance” (18, 20) and might play an important role in reducing chemotherapy-induced side effects and dose-limiting toxicities, which in turn can lead to an improved QoL as well as a reduced number of treatment terminations. In line with preclinical studies, IF and STF have been shown to decrease IGF-1 levels significantly in a healthy population (9) as well as in cancer patients (14, 21). Furthermore, Vernieri et al. recently demonstrated a favorable shift of peripheral blood cells towards enhanced intratumor Th1/cytotoxic cells and activation of antitumor immune programs through a fasting-mimicking diet in breast cancer patients (21).

Despite the great potential of therapeutic fasting in oncology and its safety proven in several small clinical trials, only a few phase II/III studies have been conducted so far, none using a 16:8h-IF regiment. Thus, in this multi-center, randomized-controlled trial we aim to assess the effects of 16:8h-IF on quality of life and especially on fatigue during chemotherapy in patients with gynecological cancers.

## Methods

### Study design and setting

This study is designed as a prospective, two-arm parallel-group, multi-center, open-label, randomized-controlled clinical trial (phase III trial). It will be conducted at the Department of Gynecology and Obstetrics of the University Medical Centre of the Johannes Gutenberg University Mainz, Germany, as well as the department of Gynecology and Obstetrics at the St. Josef’s Hospital, Wiesbaden, Germany. All data will be recorded pseudonymized. Only the principle investigator of the study, as well as the study nurses in charge will have access to the pseudonymization list in case of adverse events or trial termination.

The active fasting period will be conducted for three months during CHT. Participants will be scheduled for study visits during their routine care (d1 of each cycle) and routine laboratory visits on days 8 (and 15 in three week cycles) of each cycle during the three months of the active trial period. Additional study visits will be scheduled for nutritional counseling prior to the start of the chemotherapy as well as a follow up visit at six months (**Table 1**). QoL including fatigue assessment will be performed at baseline, each week during the 12 weeks active trial period as well as at the six months follow-up. Specific quality of life aspects regarding sleep quality and pelvic floor function will only be assessed at baseline, after three months and after six months.

**Table 1:** Visit overview and outcome measures assessed during each study visit.

Timepoint	Study period						
	t_-1_ *(3d - 4w prior to CHT)*	All cycles d_1_	All cycles d8	3-week cycles day 15	12 weeks after Start CHT ( ± 1 week)	Post surgery where applicable	6 months follow up ( ± 2 weeks)
Enrolment Eligibility screen Informed consent Randomization Allocation	XXXX						
Interventions Intermittent fasting Normal diet Nutritional counselling	XXX				→ →		Voluntary continuation of IFSwitch to IF possible
Assessments Demographics Medical history Current medication Anthropometric measurements Vital signs Physical examination CTCAE Hematogram Glucose/ketone levels Organ Blood panely-H2AX DNA damage essayImmunological responseTumor response Tumor markers Pathological response Radiological response	XXXXXXXXXXX(X)^3^	XXXXXXXXX^1^	XX^2^	X	XXXXXXXXX(X)	X	XXXXXXXX(X)
Questionnaires *Fatigue → FACIT-FS©General QoL → FACT-G© General wellbeing → EORTC QLQ-C30© TOI mental health TOI physical healthTumor specific FACT-B© FACT-O© FACT-Cx© FACT-En©Pelvic floor functionalitySleeping qualityAdherence diary Continuation/Acceptance/ Difficulties		XXXXXXXXXX^4^X^5^X^6^	XXXXXXXXX	XXXXXXXXX	XXXXXXXXXXXX →		XXXXXXXXXXX

* Some questionnaires can be calculated as subgroups from other questionnaires. Thus, only 2-3 questionnaires will need to be completed by the participants.

^1^ y-H2AX DNA damage essay will only be performed on the day of chemotherapy at cycle 1 day 1, after 6 weeks and after 12 weeks.

^2^ The immunological response will only be assessed at baseline (day of enrollment), as well as one week after chemotherapy is given at three time points (week 1, week 7 and week 13).

^3^ Radiological response will only be assessed in patients treated neoadjuvantly for ovarian/peritoneal or cancer of the fallopian tubes (response rate to neoadjuvant CHT), as well as in metastasized patients of all included tumor types.

^4^ Pelvic floor functionality will only be checked on day 1 of cycle 1; 12 weeks after the start of chemotherapy and during the 6 month follow-up.

^5^ Sleeping quality will only be checked on day 1 of cycle 1; 12 weeks after the start of chemotherapy and during the 6 month follow-up.

^6^ Adherence will only be checked via a daily diary in the intermittent fasting group.

### Recruitment and randomization

Patients will be recruited at the time of diagnosis and discussion of the treatment plan after the interdisciplinary tumor conferences of each hospital had finalized the treatment schedule. Written informed consent will be obtained from all participants prior to randomization and baseline visit. Patients can withdraw consent and effectively withdraw their trial participation at any point without any disadvantages. A screening checklist including all inclusion and exclusion criteria will be assessed by a medical doctor prior to randomization. Variable block randomization stratified by tumor entities and study site will be used to randomly assign participants to intervention or control group (1:1). Block length will be disclosed upon completion of the trial. The preparation of the randomization lists will be performed by a statistically trained researcher not involved in the recruitment, allocation, or assessment of the participants. Randomization lists will be uploaded in RedCap and cannot be viewed or changed during the trial. Group allocation will be performed over the RedCap database. Patients will be allocated after obtaining consent and completing baseline assessments to confirm eligibility.

### Eligibility criteria

Adult female patients with histologically confirmed gynecological cancers (ovarian, breast, cervical or uterine) are eligible to participate in this study. Potential trial participants will be identified through interdisciplinary tumor conferences at both study centers and potential study participation will be documented in the protocol. A complete list of inclusion and exclusion criteria can be found in **Table 2**. Exclusion criteria were chosen to minimize the risk for critically ill patients as well as to exclude diseases significantly impacting the normal metabolism.

**Table 2:** Inclusion and exclusion criteria of the study.

Inclusion criteria	
	◼ Women with histologically confirmed ovarian (including peritoneal cancer or cancer of the fallopian tubes), breast, endometrial or cervical cancer ◼ ≥ 18 years of age
	◼ BMI ≥ 19 kg/m^2^ ◼ ECOG 0-2 at baseline ◼ Anticipated life expectancy of > 6 months ◼ Planned to receive intravenous chemotherapy for at least three months ◼ Planned to receive chemotherapy including at least one topoisomerase inhibitor or alkylating agent
	◼ Ability to understand nature and effects of the trial participation
Exclusion criteria	
	◼ Simultaneous radiation therapy ◼ Received Chemotherapy in the last six months ◼ Weight loss of >10% in the last three months ◼ Type I diabetes or intensified insulin treatment
	◼ Kidney failure (creatinine above 2mg/dl), liver failure (liver enzymes above 2,5x the upper limit of normal)
	◼ Current or past eating disorders ◼ Other psychiatric diseases
	◼ Myocardial infarction, stroke or pulmonary embolism within the last 3 months
	◼ Unstable heart disease ◼ Severe internal co-morbidities
	◼ Enterostomy, short bowl syndrome
	◼ Pregnancy & breastfeeding period
	◼ Participation in another interventional trial likely to interfere with the study outcome ◼ Language difficulties

### Study intervention and control

All participants will receive a teaching by a registered dietician as well as a pamphlet on dietary tips to cope with CHT related adverse events.

#### Intermittent fasting group

To increase the feasibility of IF during CHT, the intervention group will be asked to follow an IF schedule of at least 16 h continuous fasting per day on a minimum of five days per week. This allows a disruption of the fasting due to chemotherapy-related issues or to accommodate everyday events such as birthdays or short visits in the IF schedule. We have successfully tested this fasting routine in a pilot study of healthy volunteers, where ketone bodies reached the desired concentration after 16 hours of fasting. We found IF to be safe and effective in reducing fatigue and increasing QoL even in healthy volunteers. The IF schedule was feasible in a stressful work environment and variable working hours during shift work (9). To ensure the positive benefits during the administration of CHT, participants will be required to adhere strictly to the 16h fasting period per day from two days before until one day after the administration of i.v. CHT. To prevent possible negative effects from reversing the metabolic switch to an anabolic state once eating again, no food intake will be allowed on the day of CHT. On the remaining time period of the cycle, the two days of non-fasting per week are allowed. The 16-hour continuous fasting interval can be started at any time point each day. While adhering to the same fasting schedule each day is recommended (e.g., fasting in the morning) to establish behavioral manners, this is not compulsory. Non-sugary drinks including water, tea or coffee without milk (soy milk, oat milk, cow milk etc.), as well as clear vegetable broth are allowed during the 16h fasting period. Within the non-fasting period of 8 hours per day no dietary restrictions will be given. However, a normo-caloric Mediterranean diet are recommended for all patients. Participants of the intervention group will receive a 20-minutes one-on-one counselling by a licensed dietician.

#### Control group

The control group will not be given any dietary restrictions. A normal-caloric Mediterranean diet is encouraged and a personal counseling session with a licensed dietician will be provided before starting CHT for every participant of the control group.

### Adherence

To increase compliance, all participants will be offered assistance with any problems related to fasting and adherence at each planned study visit. Furthermore, all participants will receive written information on dietary advice and the management of CHT-related gastrointestinal toxicities. Individual diet-specific questions can be discussed with a medical doctor at any time during the study in person or via electronic communication. Adherence will be measured through a specifically designed simple daily diary (three smiley system) and the hydroxybutyrate level in the blood (assessed at day 1 of every cycle). To increase long-term practicability in everyday life (e.g. shift work, family events) and to allow for adjustments to individual lifestyles, participants of the intervention group can break the IF diet for up to two days per week (five days fasting, two days off).

### Baseline characteristics

For eligibility screening and baseline assessments, data of potential trial participants including full oncologic history, vital signs, anthropometric measurements, full medical history, concomitant therapies, pre-existing adverse events from prior chemotherapy as well as full inclusion and exclusion criteria will be recorded. Once recruited for the trial, all participants will be asked to complete questionnaires measuring current QoL and baseline laboratory checks will be performed (see **Table 1**).

### Outcome measures

#### Primary outcome

Primary outcome of this study is the difference of the mean change in fatigue from baseline to last follow-up time point of the active trial period (3 months) between the IF group and the control group. A variation of up to ± 1 week for trial termination is allowed due to potential delays of CHT (e.g. due to insufficient blood values or public holidays).

Fatigue is measured after each chemotherapy treatment using the Functional Assessment of Chronic Illness Therapy-Fatigue Scale (FACIT- FS©). This questionnaire assesses cancer therapy related fatigue. It is part of the FACIT-F© questionnaire (22) which is a combination of the Functional Assessment of Cancer Therapy general core questionnaire (FACT-G©) and the FACIT-FS©. The FACIT-FS© includes 13 fatigue related questions, which are rated on a 5-point Likert scale (not at all to very much) over the last 7 days. Scores will be calculated according to published scoring algorithms.

#### Secondary outcomes

As there is only very limited availability of data regarding fasting during CHT, a broad range of secondary endpoints for future confirmatory clinical trials will be assessed. A detailed overview of the assessed outcomes at each time point is summarized in **Table 1**.

#### Quality of life

General QoL and overall health as measured by the two general wellbeing items of the European Organization For Research and Treatment Core Quality of Life Questionnaire (EORTC QLQ-C30 version 3.0© will be assessed. Furthermore, the FACT-G© questionnaire to assess cancer therapy associated QoL, as well as QoL questionnaires specific to gynecologic cancers FACT-B© (breast cancer), FACT-O© (ovarian cancer), FACT-EN© (endometrial cancer), FACT-CX© (cervical cancer)) will be completed by the patients to assess the effects of IF on QoL. The FACT-G© questionnaire forms the core of the FACT questionnaires and is complemented by the specific cancer type related questionnaire. Items relate to disease-, treatment- and condition-related QoL. Scores will be calculated according to published scoring algorithms by the FACT© association. As sleep quality and pelvic floor deficiencies are often related with gynecological cancers and CHT, these aspects will be assessed separately by validated questionnaires. Sleep quality will be examined through the Pittsburgh Sleep Quality Index, a short questionnaire consisting of 19 items, which can be summed up in seven different components of sleep quality such as sleep duration, sleep efficiency, disruptions of sleep, etc. (23). Pelvic floor deficiencies will be assessed with the German pelvic floor questionnaire, which includes items regarding sexual function, bladder and bowl disorders and associated QoL (24).

#### Feasibility and adherence

Due to the limited evidence currently available on fasting during CHT, we will assess the feasibility of the proposed IF schedule during CHT by recording adherence to the diet in a simplified daily diary (three smiley system), as well as the number of major protocol violations and discontinuations of the trial at each study visit.

#### Body weight changes and safety

To assess the safety of IF during CHT, during which many patients struggle to maintain their weight, changes in BMI will be recorded and compared between groups. Furthermore, regular laboratory assessments of blood values such as hematograms, liver and kidney function will be assessed over the study period. A detailed overview of laboratory values assessed can be found in **Appendix A**. Laboratory values will be accepted if obtained ± 1 day of the planned assessment day (e.g. one day prior to CHT).

#### Chemotherapy-related toxicities

To assess the effect of IF on chemotherapy-related toxicities (hematological and non-hematological toxicities), the incidence and severity of treatment-related adverse events (AEs) will be recorded by the Common Terminology Criteria for Adverse Events (CTCAE, v5.0). Laboratory measures focused on hematological toxicities, renal and liver functions as well as specific biomarkers for metabolism and tumor immunology (see **Table 1**) will be analyzed during the whole study period.

#### Tumor response

Effects of IF on tumor response between intervention and control group will be compared by tumor specific markers (e.g. CA 125, CA 15-3, CEA, SCC, LDH), pathological response rates after neoadjuvant treatment as well as standardized radiologically evaluation with RECIST 1.1 criteria in metastasized cancers. Imaging will be obtained during routine clinical follow-up and will not be performed specifically for this trial. Radiological responses will be evaluated by a single radiologist blinded to the group allocation of the patient. Pathological responses will be extracted from official medical records after surgery. Pathologists will not be aware of trial participation or group allocation.

#### Immunological response

In a recent study cyclic fasting during chemotherapy showed to downregulate peripheral blood immunosuppressive myeloid cells while increasing activated/cytotoxic cells (21). Thus, we plan to perform a multicolor flow cytometry 7 days after the administration of chemotherapy during the active trial period at 1 week, 7 weeks and 13 weeks. As the immunological changes are dependent on the day CHT is given, time points will be adjusted +/- 1 week depending on the days CHT is administered (e.g. in case of delayed CHT or public holidays). The blood samples will be stored in X-VIVO-medium with 10% DMSO (1 x 10^7^ cells/mL) at -80°C. Peripheral blood mononuclear cells (PBMCs) will be isolated from venous whole blood of the patients by gradient centrifugation. 10 ml blood will be separated by Ficoll using Leukosep tubes (Greiner) after lysis of erythrocytes (RBC lysis solution, Qiagen). The detection of leukocyte surface markers will be performed by using fluorochrome conjugated antibodies specific for CD3, CD8, CD14, CD15, CD25, CD56, CD69, HLA-DR, PD-L1 and PD1. Monocytes will be indicated by CD14 expression, PMN-MDSCs by CD15 expression, M-MDSCs by CD14 expression but HLA-DR negativity, PD-L1 positive immunosuppressive monocytes by CD14 and PD-L1 expression, T cells by CD3 expression and cytolytic NK cells by CD16 and CD56^dim^ expression of CD3 negative cells (21). To detect further immunological reactions, the cytokines CCL2, G-CSF, IL-6 and IL-8 will be quantified by ELISA (R&D Systems).

#### Cell damage

Recent studies demonstrated a protective effect of short term fasting on healthy peripheral blood cells during chemotherapy (14). Thus, we aim to assess DNA damage in peripheral blood mononuclear cells (PBMCs) using the level of y-H2AX intensity after the administered chemotherapy (in case of more than one chemotherapy, the blood will be drawn right after the first chemotherapy). Blood will be drawn at the first day of CHT, six weeks and 12 weeks after the start of the CHT. As the cell damage is dependent on the day CHT is given, time points will be adjusted +/- 1 week depending on the days CHT is administered (e.g. in case of delayed CHT or public holidays). Blood samples will be stored in X-VIVO-medium with 10% DMSO (1 x 10^7^ cells/mL) at -80°C.

When DNA is damaged, the histone H2AX gets phosphorylated at Ser139 in the area of damage. For detection of phosphorylated H2AX (γH2AX), PBMCs, isolated as described above, will be cytocentrifuged onto glass slides (2 x 10^4^ cells/slide), fixed by paraformaldehyde, treated with 0.1% Triton X100 and stained immunocytologically by using a specific Flour 488 labeled antibody (Abcam). A counterstain will be performed by DAPI and the slides will be evaluated by laser scanning microscopy. For detection of γH2AX in tumor tissue, formalin fixed and paraffin embedded tumor tissue will be immunohistochemically stained by using an anti-γH2AX (Ser 139) antibody (Cell Signaling). The staining will be performed with EnVision™ staining kit (Dako) according to the manufacturer’s instructions. The evaluation will be performed by light microscopy.

#### Long-term effects

Long-term effects after termination of the intervention on fatigue, QoL, laboratory changes and rate of voluntary continuation of IF will be assessed in a single follow-up visit at 6 months (+/- 2 weeks) after completion of CHT. If tumor status is obtained through routine clinical follow-up, it will be assessed through imaging (when appropriate) and biomarker response.

### Methods against bias

#### Selection bias

Potential trial eligibility will be assessed during an interdisciplinary tumor conference and all patients will be approached consecutively for trial participation. Final eligibility will be determined during the consultation for obtaining informed consent for the planned CHT. For all eligible non-participants, the reason for non-participation will be documented. Moreover, eligible participants and non-participants will be compared regarding age, cancer type, ECOG and FIGO stage to identify potential selection processes.

#### Detection bias

All outcomes will be predefined, and the majority of outcome parameters are assessed by clear definitions that do not leave much room for interpretation. Subjective outcomes are documented by the participants themselves and cannot be influenced by the study staff.

### Statistical analysis

Patients will be randomized to the intervention or control group (1:1) stratified by tumor entity and study site. The primary endpoint, the difference of the mean change of fatigue over a period of three months as measured by the FACIT-FS© score, will be compared between both groups using mixed models. Confounders such as age, type of CHT, neoadjuvant/adjuvant vs metastatic treatment, the Eastern Cooperative Oncology Group performance status (ECOG), histological tumor grade, and Fédération Internationale de gynécologie et d’Obstetrique (FIGO) -stage will be included in the mixed models. Laboratory data, as well as QoL questionnaire-based outcomes will also be assessed using mixed models. Missing data for the primary outcome as well as secondary outcomes with the potential to become main outcomes in subsequent larger trials will be imputed. Where possible, missing questions from FACT^©^ questionnaires will be prorated using the average of the other answers in the subscale (prorated subscale score = [sum of the item scores]*[N of items in subscale]/[N of items answered]) as long as more than 50% of the items are answered. Data will be imputed by drawing values from the distribution of the control group (jump to reference).

A comprehensive summary will be given for primary and secondary endpoints, including absolute and relative frequencies for categorical and binary variables as well as mean and standard deviation or median and interquartile range and minimum-maximum for all data with at least interval scale level. Pathological response rates after neoadjuvant CHT will be compared using the Chi square test. Radiological response rates (complete response, partial response, stable disease, and progressive disease) will be compared between the arms at 3 months and 6 months using the Mann-Whitney-U-tests. The occurrence of any adverse event as well as the occurrence of grade 3 or higher adverse events will be compared between both groups using the Chi square test. And a list of immunological and metabolic parameters will be compared between both groups using the Mann-Whitney-U test. A paired Wilcoxon test will be used to compare the change in BMI over three months between both groups.

Analyses will be performed as (1) modified intention-to-treat (ITT) population including all patients randomized who have at least received the first chemotherapy cycle. Patients will be analyzed in the group they were randomized to; (2) per-protocol (PP) sets, including all patients who have at least completed one/two/three or all chemotherapy cycles without major protocol violations. Safety will only be assessed in the per-protocol subset. Subgroup analyses will be performed by tumor type and type of CHT regime for groups with an adequately high number of participants. Furthermore, the parameters will be displayed graphically, and appropriate descriptive statistics will be employed. The significance level will be bilaterally set at 5%. 95% confidence intervals will be reported where appropriate. Due to the exploratory nature of secondary outcomes, no adjustments will be made for multiple testing.

### Sample size

Sample size calculations were based on a recently published study by Bauersfeld et al. assessing short-term fasting during chemotherapy of breast and ovarian cancer patients (13). A sample size of 48 patients per group is necessary to detect a mean difference of 9.1 points on the FACIT-FS^©^ scale with a standard deviation of 13.7 points and a power of 90% and α=0.05 with a two-sided independent t-test. To account for possible drop-outs, a sample size of 55 per group at baseline is planned.

### Data monitoring, interim analysis and trial termination

Data will be collected on a personalized case report form at the predefined time points depicted in **Table 1**. Data will be entered in a protected database (RedCap). Only authorized members of the IFAST study team will be allowed to enter, store and access participant data. Paper-based data will be stored at the department of obstetrics and women’s health at the University Medical Center Mainz. Clinical routine data assessments will be stored in the respective patient’s clinical files at the University Medical Center Mainz and St. Josef’s Hospital.

The safety of IF has been proven in many smaller studies, including our pilot trial (9) for healthy people as well as patients under chemotherapy (25). No severe adverse events were observed; thus, no interim analysis is planned. The trial can be terminated by the patient at any time without the risk of negative consequences by withdrawing consent. The discontinuation of the intervention can be decided upon by the investigator due to medical reasons, including the reduction of the BMI to <18 kg/m^2^, acute severe CHT induced side-effects requiring hospitalization or acute, severe non-chemotherapy related medical illnesses requiring hospitalization. Patients terminating the intervention will be asked to fill out the questionnaires outside of the study protocol.

A data monitoring committee consisting of a medical doctor and a study nurse will be overseeing the study visit schedules. All investigators will be obliged to report any changes or deviations of the protocol to the data monitoring committee. Any serious adverse events will be reported to the data monitoring committee and discussed with the principal investigator within 48 hours of occurrence. The termination of the trial for the participant can be decided upon based on the criteria mentioned above by the principal investigator. The termination of the whole trial can be determined by the principal investigator together with the data monitoring committee due to unexpected serious adverse events based on the intervention or due to failure to recruit the planned number of patients.

### Strengths

This is the first randomized-controlled, multi-center trial (phase II-III) assessing the effects of IF during CHT in gynecological cancer patients. With a planned sample size of 110 patients, it is comparable to other clinical trials with similar designs. The eligibility criteria are broad, allowing patients with various clinical cancers to enter the trial. This will allow for a better generalizability of the results to clinical practice. Due to the frequent checkups during active CHT, the changes in fatigue and QoL will be recorded in relation to the days from the last administration of CHT. This will allow us to evaluate the changes in fatigue and QoL, which are often improving towards the end of each chemotherapy cycle. Furthermore, the IF concept used in this trial allows for flexibility in food choices and personalized time schedules for each patient. This might increase compliance compared to previously assessed fasting mimicking diets or longer fasting periods of 72h (14, 21).

### Limitations

With increased generalizability due to the inclusion of different cancer types and CHTs (+/- immunotherapy), comes a greater heterogeneity in trial results. Thus, the secondary endpoints of this clinical trial are only exploratory. However, these exploratory results are essential for generating new hypotheses, detecting meaningful endpoints and better sample size estimations for future clinical trials. Depending on the distribution of included patients, the data might be of limited significance for some cancer types or CHT regimes. Especially, the type of cancer therapy greatly influences the magnitude of the experienced QoL impairment. Thus, in this trial only i.v. CHTs including at least one alkylating agent or topoisomerase inhibitor are allowed. Furthermore, we assess the difference in mean change with regards to CHT regimes to minimize the effects of different CHT regiments on the results. In addition, many side effects of CHT such as fatigue, limited concentration abilities, physical weakness or gastrointestinal symptoms are common in most i.v. CHTs. Thus, the results of this trials should be generalizable especially with regards to QoL independent of the distribution of patients. A confounding factor of our study could be that the fasting period parallels over the cause of the year with the hay fever and summer season, which in itself may lead to increased fatigue in some subjects, diminishing possible effects (26). However, by comparing long-term effects we aim to limit intermittent periods of increased fatigue and expect the fatigue induced by CHT to exceed allergy related symptoms.

## Discussion

This is the first randomized-controlled, multi-center, clinical trial assessing the effects of 16:8h-IF on the QoL and fatigue during CHT in gynecological cancer patients. Due to the currently limited clinical evidence several secondary endpoints, including the incidence of adverse events during chemotherapy, rate of pathological remissions after neoadjuvant treatment, progression of disease in imaging, the peripheral cell damage and change in peripheral immunological profile will be assessed exploratively and will provide new insights about the mechanisms and potential of IF during CHT.

Many studies have shown positive effects on general health and quality of life through various forms of diet (27, 28). While some diets rely on caloric restrictions to achieve positive effects like weight loss and associated metabolic improvements, others include IF aiming to positively influence the body by achieving a metabolic switch (ketogenesis). Current trials evaluated IF in heterogeneous study populations including healthy adults (28, 29) fasting during Ramadan (27, 30, 31), competitive athletes, oncologic patients during systemic chemotherapy (17, 32–34) as well as in patients suffering from obesity or cardiovascular diseases (35–38), with partly controversial results. Overall, the demonstrated effects of fasting were influenced by different intervals of fasting, varying fasting periods and had shown to be highly dependent on the type of IF and the specific population. The normal metabolism needs several weeks to sustainably switch energy production to ketogenesis; thus, an active trial period of three months (12 weeks) was chosen for this trial.

Various fasting regimes were promoted for IF, including alternating fasting days or fasting periods between 10-20 hours, while true fasting often requires more than 24 hours of fasting. In direct comparison to true fasting, IF showed a greater reduction in subjective mental fatigue in a study including 17 healthy, non-obese females under 50 years of age (39). A currently ongoing phase II single-group clinical trial assessing IF in breast cancer patients treated with i.v. CHT prior to surgery chose the use of a 14 hours fasting period followed by 10 hours eating period (NCT05327608). The trial is unfortunately currently on hold due to staffing issues. However, to achieve a reliable metabolic switch, at least 16 hours of food restriction is recommended (40, 41). Thus, flexible fasting intervals of 16 continuous hours per day including sleeping phases were chosen for this trial to assure ketogenesis. In a clinical trial by Anton et al. a similar fasting schedule (16:8) was chosen to assess QoL in overweight older patients. The mentioned study detected an improvement in QoL after 4 weeks (42), while our study comprises three months, ensuring short- and long-term observations. To increase the adherence a deviation of the 16:8 fasting schedule will be allowed at a maximum of two days per week with exception of the 2 days prior to and after CHT. This flexibility is offered to increase adherence and allow for spontaneous events in everyday life (such as birthday parties or family dinners), which are an integral part of QoL.

Generally, the feasibility of fasting schedules is of great importance to the success of any dietary change. While IF is thought to be a very feasible diet in everyday life, especially the first 2 weeks can be challenging and are often associated with side effects such as headache and increased fatigue or thoughts about food. These changes were also seen in our pilot trial (9). However, all probands described improvements after only a few days and thoughts about the fasting process itself decreased within the trial period. Overall, the feasibility of the fasting schedule was described as high, which was also shown in the good adherence throughout the trial. However, patients receiving CHT are challenged with other obstacles such as side effects of CHT. Thus, especially in the beginning it is expected to be harder for the intervention group to stick to the proposed fasting schedule. Nevertheless, due to the flexibility of the proposed fasting schedule with possible adjustment to each patients personal preferences (fasting periods can be chosen freely and changed throughout the trial) and allowed days of the fasting regimen (e.g. for birthdays or other important life events) we expect the fasting intervention to be manageable for 12 weeks. To support patients during the initial transition to IF, the expected side effects of IF and CHT are discussed in the personal counselling with a licensed dietician, along with possible methods to counteract them while practicing IF. Furthermore, individual guidance to determine the most feasible fasting schedule depending on the patients preferences is provided during the dietary counselling and medical personal is available to support patients experiencing difficulties with the IF schedule throughout the trial.

Fatigue is a common side effect of many CHTs. In a cross-sectional study of breast cancer patients clinically meaningful fatigue was seen in almost 50% of patients under CHT (43). Unfortunately, so far fatigue cannot be treated effectively with medication and only few measures such as physical activity are known to positively influence chemotherapy-associated fatigue (44, 45). Thus, it is of utmost importance to evaluate potential treatment options to reduce fatigue during CHT, without impairment of the oncologic treatment. With demonstrated positive effects on fatigue through IF in healthy patients (9), we aim to confirm the hypothesis that IF following can decrease fatigue during CHT.

To ensure the safety and exclude negative effects on the anti-tumor activity of CHT through the periodic activation of the ketogenic metabolism for energy production by IF, several laboratory values reflecting the general health in blood samples will be drawn regularly. Several studies suggest significant changes in laboratory parameters including a decrease in blood glucose levels and HbA1C (30, 37), serum cholesterol, liver profiles (46) and oxidative stress (2, 3). Furthermore, growth factors such as IGF-1 have been associated with decreased side effects during systemic chemotherapy (47, 48). Thus, we aim to explore the changes in meaningful disease-associated blood values caused by IF, such as IGF-1, interleukin 6 and 8 as well as tumor markers. Finally, regular basic values including liver and kidney function are checked to ensure the safety of IF during CHT.

Recently, Vernieri et al. demonstrated a change in peripheral blood mononuclear cells caused by a fasting mimicking diet during chemotherapy in breast cancer patients. They were able to show a downregulation of immunosuppressive myeloid cells while simultaneously activating cytotoxic cells (21). Unfortunately, only 20% of patients were able to comply with the fasting schedule for a total of eight cycles. Similar results in compliance were seen in a trial by de Groot et al. using a fasting mimicking diet during CHT in patients with breast cancer (14). Interestingly, they were able to show a reduction in peripheral cell damage (measured by the level of y-H2AX) in those compliant with the diet, indicating a protection of healthy cells without limiting the cytotoxic effects on cancer cells. So far, no evidence exists assessing the influence on immunological changes and peripheral cell damage during CHT in patients with gynecological cancers. Thus, we aim to assess these changes in our cohort.

In summary, the IFAST trial is of great importance to evaluate the effect of IF on fatigue during CHT to offer patients an alternative of actively influencing their well-being during cancer treatment. Furthermore, this trial will help to gain more insight on the effects of fasting on the immune system and efficiency and tolerance of chemotherapy, despite being exploratory.

## Data availability statement

The datasets for this article are not publicly available due to concerns regarding participant/patient anonymity. Requests to access the datasets should be directed to the corresponding author.

## Ethics statement

The study was registered at the German Register of Clinical Trials (DRKS) on the 8th of March, 2023 (ID: DRKS00031429). The trial will be conducted in accordance with the “Ethical principles for medical research involving human subjects” of the current version of the Declaration of Helsinki. All data will be recorded and analyzed pseudonymized and treated confidentially, by authorized study personal only. Ethics approval was obtained from the Ethics Committee of the state medical association of Rheinland-Pfalz, Germany (2020-15136_5) and the state medical association of Hessen, Germany (2023-3321-zvBO) based on protocol version 4.0 (08.01.2023). Written informed consent will be obtained from all participants and participants are free to leave at any time without giving reasons for their decision. If at any time the physical and mental health becomes jeopardized due to partaking in the study, the participation will be terminated immediately (see trial termination). Patients will be allowed to refuse additional blood draws to assess the change in PBMCs, without exclusion from the trial.

## Author contributions

Study conception and design: KAn, MWS, WB, MS, AH, BB-S, ALu, SS, DP; methodology: MWS, KAn, WB, SG, LW, AM-K, MR; Data collection: MWS, KAn, BB-S, ALe, HH, DP, LW, ALu, SG; Statistical planning: MWS, SS; Patient counselling: MWS, KAn, LW, DP, MS, ALu, VL, SK, MB, RS, BB-S, ALe, KAl; Project administration and supervision: MWS, MS, KA, AH, BG; Drafting of manuscript: MWS, KAn, SS, WB, SG, HH, DP; Critical revision: AH, BG, MS, RS, MB, BB-S, ALe, KAl, Alu, MR, AM-K, SK, VL, LW. All authors contributed to the refinement of the study protocol and have approved the final manuscript.
