# Supplementary material for: Effects of intermittent fasting on quality of life tolerance of chemotherapy in patients with gynecological cancers: study protocol of a randomized-controlled multi-center trial
Source: Front Oncol. 2023 Jul 19;13:1222573. doi: 10.3389/fonc.2023.1222573 (PMC10396395; doi:10.3389/fonc.2023.1222573)
Supplement: Supplementary file 1 [file Table_1.docx]

**Appendix**

**Appendix A** Laboratory values and time points

| **Panel** | **Laboratory values** | **Timepoint** | | | | | |
| --- | --- | --- | --- | --- | --- | --- | --- |
|  |  | t-1 | Day 1 of each cycle | Day 8/15 of each cycle | Additionally after 6 weeks | After 12 weeks | Follow-up |
| **Blood count** |  |  |  |  |  |  |  |
|  | Differential blood count | X | X | X |  | X | X |
|  | Reticulocytes | X |  |  | X | X | X |
| **Organ function** |  |  |  |  |  |  |  |
|  | Kreatinin | X | X |  |  | X | X |
|  | Aspartate aminotransferase (GOT) | X | X |  |  | X | X |
|  | Alanin aminotransferase (GPT) | X | X |  |  | X | X |
|  | Gammaglutamyl transferase (yGT) | X | X |  |  | X | X |
|  | Bilirubin | X | X |  |  | X | X |
|  | Thyroxin (fT4) | X |  |  |  | X | X |
|  | Triodothyronine (fT3) | X |  |  |  | X | X |
|  | Thyroid stimulating hormone (TSH) | X |  |  |  | X | X |
| **Electrolytes** |  |  |  |  |  |  |  |
|  | Sodium | X |  |  |  | X | X |
|  | Potassium | X |  |  |  | X | X |
|  | Magnesium | X |  |  |  | X | X |
|  | Calcium | X |  |  |  | X | X |
|  | Chloride | X |  |  |  | X | X |
| **Metabolism** |  |  |  |  |  |  |  |
|  | Hemoglobin A1c (HbA1c) | X |  |  |  | X | X |
|  | High-density lipoprotein (HDL) | X |  |  |  | X | X |
|  | Low-density lipoprotein (LDL) | X |  |  |  | X | X |
|  | Triglyceride | X |  |  |  | X | X |
|  | Ferritin | X |  |  |  |  |  |
|  | Transferrin saturation | X |  |  |  |  |  |
|  | Albumin | X |  |  |  | X | X |
|  | Uric acid | X |  |  |  | X | X |
|  | Urea | X |  |  |  | X | X |
|  | 3-hydroxybutyrat |  | X |  |  |  |  |
| **Other** |  |  |  |  |  |  |  |
|  | C-reactive protein | X |  |  | X | X | X |
|  | Interleukin-6 | X |  |  | X | X | X |
|  | Interleukin-8 | X |  |  | X | X | X |
|  | Insulin-line growth factor 1 (IGF-1) | X |  |  | X | X | X |
| **Tumor markers** |  |  |  |  |  |  |  |
|  | Carcinoembryonic antigen (CEA) (breast/ovarian/peritoneal/fallopian tube/cervical cancer) | X |  |  |  | X | X |
|  | Carcinoma Antigen 15-3 (breast cancer) | X |  |  |  | X | X |
|  | Carcinoma Antigen 125 (ovarian/peritoneal/fallopian tube cancer) | X |  |  |  | X | X |
|  | Squamous Cell Carcinoma Antigen (cervical cancer) | X |  |  |  | X | X |
|  | Laktate dehydrogenase | X |  |  |  | X | X |
|  |  |  |  |  |  |  |  |
